# Unlocking Synergistic Photo-Fenton Catalysis with Magnetic SrFe_12_O_19_/g-C_3_N_4_ Heterojunction for Sustainable Oxytetracycline Degradation: Mechanisms and Applications

**DOI:** 10.3390/nano15110833

**Published:** 2025-05-30

**Authors:** Song Cui, Yaocong Liu, Xiaolong Dong, Xiaohu Fan

**Affiliations:** 1 International Joint Research Center for Persistent Toxic Substances (IJRC-PTS), School of Water Conservancy and Civil Engineering, Northeast Agricultural University, Harbin 150030, China; s220101026@neau.edu.cn (Y.L.); b220101012@neau.edu.cn (X.D.); b220101018@neau.edu.cn (X.F.); 2 Research Center for Eco-Environment Protection of Songhua River Basin, Northeast Agricultural University, Harbin 150030, China

**Keywords:** oxytetracycline, photo-Fenton, SrFe_12_O_19_/g-C_3_N_4_, nitrogen defects

## Abstract

The widespread contamination of aquatic environments by tetracycline antibiotics (TCs) poses a substantial threat to public health and ecosystem stability. Although photo-Fenton processes have demonstrated remarkable efficacy in degrading TCs, their practical application is limited by challenges associated with catalyst recyclability. This study reports the development of a novel magnetic recoverable SrFe_12_O_19_/g-C_3_N_4_ heterostructure photocatalyst synthesized via a facile one-step co-calcination method using industrial-grade precursors. Comprehensive characterization revealed that nitrogen defects and the formation of heterojunction structures significantly suppress electron (e^−^)–hole (h^+^) pair recombination, thereby markedly enhancing catalytic activity. The optimized 7-SFO/CN composite removes over 90% of oxytetracycline (OTC) within 60 min, achieving degradation rate constants of 0.0393 min^−1^, which are 9.1 times higher than those of SrFe_12_O_19_ (0.0043 min^−1^) and 4.2 times higher than those of g-C_3_N_4_ (0.0094 min^−1^). The effectively separated e^−^ play three critical roles: (i) directly activating H_2_O_2_ to generate ·OH radicals, (ii) promoting the redox cycling of Fe^2+^/Fe^3+^ ions, and (iii) reducing dissolved oxygen to form ·O_2_^−^ species. Concurrently, h^+^ directly oxidize OTC molecules through surface-mediated reactions. Furthermore, the 7-SFO/CN composite exhibits exceptional operational stability and applicability, offering a transformative approach for scalable photocatalytic water treatment systems. This work provides an effective strategy for designing efficient and recoverable photocatalysts for environmental remediation.

## 1. Introduction

Antibiotics are widely utilized in animal husbandry to promote growth and prevent disease. However, the prolonged and inappropriate use of antibiotics has resulted in a range of adverse consequences. Residual antibiotics in the environment can induce the emergence and spread of antibiotic resistance genes (ARGs) and antibiotic-resistant bacteria (ARB) [1,2,3]. Additionally, these residues can adversely affect the growth and development of aquatic organisms such as algae and fish [4]. Observational, clinical, and epidemiological studies have increasingly demonstrated that antibiotic exposure may alter the composition of the host’s intestinal microbiome, potentially leading to gastrointestinal diseases [5]. Globally, antibiotic consumption continues to rise. According to Van Boeckel et al., it is projected that between 2010 and 2030, the global usage veterinary antimicrobial drugs will increase by 67% [6]. Only a minor fraction of antibiotics are metabolized or absorbed by organisms, while the majority are released into aquatic environments via various pathways [7]. Traditional water treatment technologies often fail to completely eliminate residual antibiotics from water [8,9,10], resulting in their presence in diverse water bodies worldwide, including drinking water supplies. The potential threat posed by antibiotics to aquatic ecosystems and human health is significant and cannot be underestimated [11].

Oxytetracycline (OTC), a commonly detected antibiotic in environmental matrices, is extensively utilized in animal husbandry and aquaculture owing to its cost-effectiveness and therapeutic potency [12]. As an eco-friendly advanced oxidation process, the heterogeneous photo-Fenton reaction has been established as a robust methodology for OTC elimination [13,14,15]. This technology employs solid-phase catalysts capable of generating photogenerated electrons (e^−^) and holes (h^+^) upon light irradiation. These charge carriers mediate hydrogen peroxide (H_2_O_2_) decomposition to yield reactive oxygen species (ROS) or directly engage in contaminant breakdown. Consequently, this catalytic mechanism enables either partial detoxification through molecular transformation or complete mineralization of pollutants. In contrast to homogeneous counterparts, the heterogeneous photo-Fenton system demonstrates enhanced hydroxyl radical (OH) production efficiency while circumventing operational constraints such as mandatory acidic conditions and iron-containing sludge generation inherent to conventional Fenton processes [16,17].

The development of high-performance catalysts constitutes a pivotal component in advancing heterogenous photo-Fenton systems [15]. Graphitic carbon nitride (g-C_3_N_4_), a metal-free semiconductor, has garnered significant attention as a promising functional material for photocatalytic applications [18]. This polymeric material demonstrates remarkable advantages, including the utilization of earth-abundant constituent elements, non-toxic properties, facile synthesis methods, and excellent chemical stability. Nevertheless, intrinsic limitations persist, particularly regarding restricted visible-light harvesting efficiency, limited specific surface area, and high recombination rates of photogenerated charge carriers [19]. The construction of interface heterojunction using complementary semiconductors represents an effective strategy to enhance the photocatalytic performance of g-C_3_N_4_ [20,21,22]. In semiconductor physics, heterojunctions are mainly classified into type I, type II, and type III according to their band structures [23]. Type I heterojunction facilitates efficient electron–hole pair separation through direct band contact and built-in unidirectional charge transfer mechanism, making it a focal point in the field of photocatalysis.

Ferrites demonstrate inherent magnetic responsiveness, facile synthesis pathways, abundant precursor availability, and biocompatible characteristics, properties that have garnered substantial scientific interest in advanced photocatalytic pollutant degradation [24]. These materials exhibit exceptional compatibility with g-C_3_N_4_ for constructing heterojunction catalytic architectures to optimize photo-Fenton performance. Representative studies include Wang et al.’s development of ErFeO_3_/porous g-C_3_N_4_ nanosheets [25] and Sun et al.’s fabrication of MnFe_2_O_4_/g-C_3_N_4_ composites [26], both demonstrating superior OTC degradation efficiency in photo-Fenton systems. SrFe_12_O_19_, a narrow bandgap ferrimagnetic material, is characterized by exceptional chemical stability, corrosion resistance, and pronounced ferromagnetic behavior [27], rendering it particularly suitable as a magnetic substrate for catalyst preparation [28]. Composites that integrate SrFe_12_O_19_ with transition metal dichalcogenides (e.g., MoS_2_) and bismuth-based semiconductors (e.g., BiOCl, Bi_2_O_3_) [27,28,29] demonstrate efficient contaminant removal while retaining retrievable magnetism, enabling sustainable reuse cycles.

A critical knowledge gap persists regarding the systematic investigation of SrFe_12_O_19_/g-C_3_N_4_ composite’s catalytic performance and mechanistic behavior in photo-Fenton-mediated organic pollutant degradation. Building upon established scientific foundations, we successfully engineered SrFe_12_O_19_/g-C_3_N_4_ heterostructures through a facile one-step co-calcination methodology. The objectives of this study are to (1) synthesize and characterize the SrFe_12_O_19_/g-C_3_N_4_ heterojunction catalyst using advanced techniques, systematically analyzing its surface morphology, crystal structure, optical properties, and related physicochemical attributes; (2) quantitatively assess the OTC removal efficiency under diverse aqueous matrices while evaluating the catalyst’s recyclability and magnetic separation performance; (3) elucidate the interfacial charge transfer mechanisms and demonstrate that type I heterojunctions and surface nitrogen defects synergistically enhance the separation and migration of electron–hole pairs; and (4) identify transformation byproducts through mass spectrometry analysis and propose plausible degradation pathways. These findings provide critical insights for developing cost-effective magnetic photocatalysts for practical water treatment applications.

## 2. Materials and Methods

### 2.1. Reagents

Strontium ferrite was commercially acquired from Xingkaiyue Biotechnology Co., Ltd. (Shenzhen, China). Melamine and oxytetracycline were procured from Aladdin (Shanghai, China). H_2_O_2_ and other solvents were commercially sourced from Sinopharm Chemical Reagent Co., Ltd. (Shanghai, China). The characteristic and CAS number of all chemical reagents are comprehensively detailed in Table S1 (Supplementary Materials). All reagents were certified as analytical grade purity and were employed without further purification throughout the experimental procedures.

### 2.2. Preparation of SrFe_12_O_19_/g-C_3_N_4_ Photocatalysts

**Figure 1:**
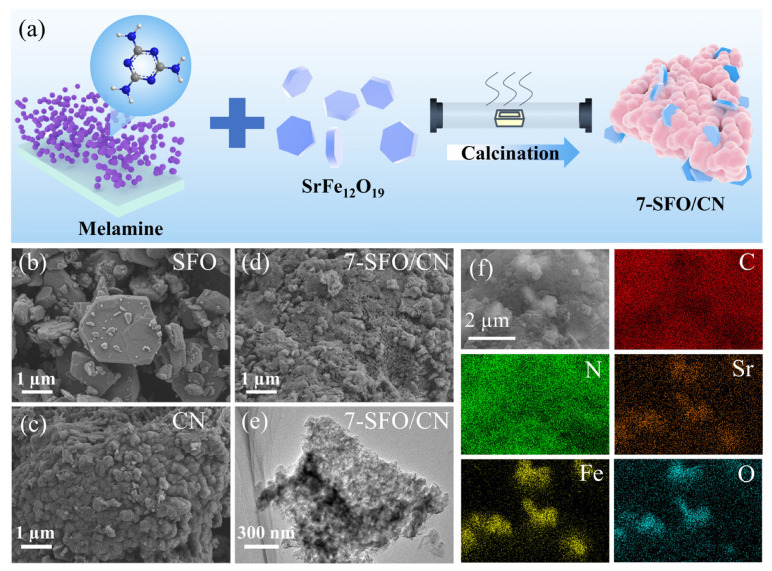
(**a**) The preparation process of SrFe_12_O_19_/g-C_3_N_4_ composite. The SEM images of (**b**) SFO, (**c**) CN, and (**d**) 7-SFO/CN. (**e**) The TEM images of 7-SFO/CN. (**f**) The SEM-EDS mapping of 7-SFO/CN.

The synthetic protocol for SrFe_12_O_19_/g-C_3_N_4_ heterostructures is schematically illustrated in Figure 1a. Specifically, 0.25 g, 0.35 g, and 0.45 g of SrFe_12_O_19_ were individually weighed and homogeneously mixed with 5.00 g of melamine to achieve mass ratios of SrFe_12_O_19_ to melamine at 5%, 7%, and 9%, respectively. The mixtures were subsequently transferred into alumina crucibles and heated at a rate of 5 °C/min to 550 °C, followed by annealing at this temperature for 2 h. The resulting materials were ground into powders and denoted as 5-SFO/CN, 7-SFO/CN, and 9-SFO/CN, respectively. Pure g-C_3_N_4_ was synthesized under the same conditions using melamine as the precursor and labeled as CN. The commercially purchased SrFe_12_O_19_ was designated as SFO.

### 2.3. Characterization

The microstructures of the synthesized catalysts were characterized using a scanning electron microscope (SEM). The elemental composition of 7-SFO/CN was analyzed by energy dispersive spectroscopy coupled with SEM (SEM-EDS). High-resolution transmission electron microscopy (HR-TEM) was employed to examine the microstructure of 7-SFO/CN. A BET analyzer was used to determine the specific surface area, pore size distribution, and N_2_ adsorption–desorption isotherm of the catalyst. The crystal structures of the synthesized photocatalysts were investigated using X-ray diffraction (XRD) and Raman spectroscopy (Raman). The chemical compositions and valence states of the samples were evaluated by X-ray photoelectron spectroscopy (XPS). The light absorption properties of the catalysts were studied via ultraviolet–visible diffuse reflectance spectroscopy (UV-vis DRS). Electrochemical impedance spectra (EIS) were recorded using an electrochemical workstation. Photoluminescence (PL) spectra were measured using a spectrometer. Electronic paramagnetic resonance (EPR) spectroscopy was utilized to detect ROS. High-performance liquid chromatography-mass spectrometry (HPLC-MS) was applied to identify intermediate products during OTC degradation. Detailed characterization methods for EIS, HPLC-MS, and EPR are provided in Text S1 (Supplementary Materials).

### 2.4. Photo-Fenton Degradation Test

The photo-Fenton reaction activity of the synthesized photocatalysts was evaluated using a 10 mg/L OTC solution. Specifically, 80 mL of the OTC solution was introduced into a beaker, and 0.02 g of the catalyst was added under gentle stirring. Adsorption–desorption equilibrium was achieved by stirring the mixture in the dark for 30 min. Subsequently, the mixture was irradiated using a 300 W xenon lamp equipped with a 420 nm cutoff filter (wavelength range: 420–780 nm). During the reaction, 5 mL samples were withdrawn every 10 min using a disposable syringe. The catalyst was then separated via filtration through a 0.22 µm mixed cellulose ester (MCE) water-based filter membrane, and the remaining OTC concentration was promptly measured at 355 nm using a UV–visible spectrophotometer. To ensure reproducibility and reliability, all degradation experiments were repeated three times. For further details, please refer to Text S2.

## 3. Results

### 3.1. Morphology and Structural Characterization

As illustrated in Figure 1b–e, the morphologies and microstructures of CN, SFO, and 7-SFO/CN were systematically characterized using SEM and TEM. SFO presented an irregular polyhedral morphology with particle sizes ranging from hundreds of nanometers to several micrometers, and significant aggregation was observed among individual SFO particles (Figure 1b). In contrast, CN displayed a compact microstructure composed of dense layered structures (Figure 1c), which aligns well with the characteristic morphology of g-C_3_N_4_ synthesized via the thermal polymerization of melamine [21]. As shown in Figure 1d, the SEM image of 7-SFO/CN did not clearly indicate the successful formation of the composite; however, the TEM image provided clear evidence of the coexistence of g-C_3_N_4_ and SrFe_12_O_19_ (Figure 1e and Figure S1). Furthermore, SEM-EDS mapping (Figure 1f) confirmed the presence of SrFe_12_O_19_ particles, approximately 2 µm in diameter, embedded within the g-C_3_N_4_ matrix. Notably, the aggregation degree of SrFe_12_O_19_ in 7-SFO/CN was significantly reduced, thereby enhancing carrier separation efficiency and photocatalytic activity [30].

**Figure 2:**
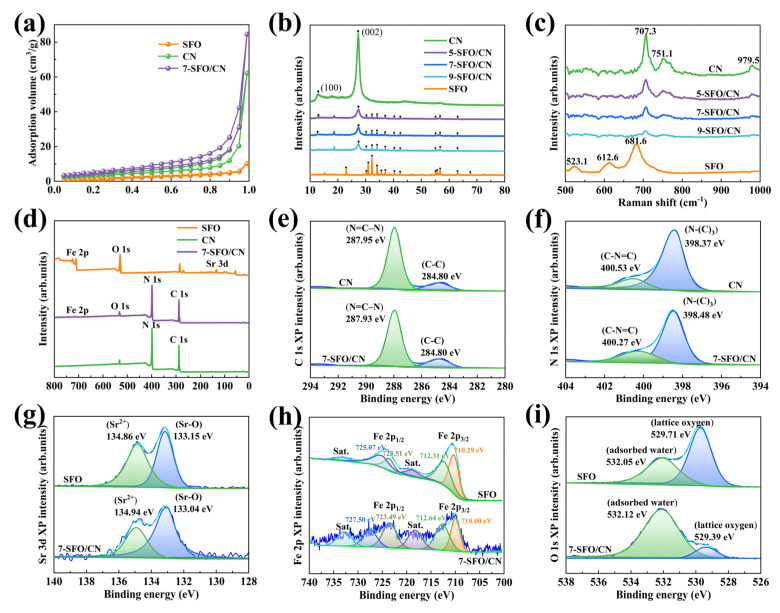
(**a**) N_2_ adsorption-desorption curves of SFO, CN, and 7-SFO/CN. (**b**) The XRD patterns and (**c**) Raman spectroscopy of SFO, CN, and SrFe_12_O_19_/g-C_3_N_4_ composites. (**d**) Survey XPS and high-resolution XPS spectra of (**e**) C 1s, (**f**) N 1s, (**g**) Sr 3d, (**h**) Fe 2p, and (**i**) O 1s of SFO, CN, and 7-SFO/CN.

Figure 2a presents the N_2_ adsorption–desorption isotherms of SFO, CN, and 7-SFO/CN. All three isotherms are classified as type IV. Additionally, the pore size distribution depicted in Figure S2 confirms that the studied materials are predominantly mesoporous. Notably, 7-SFO/CN exhibits a higher mesopore content compared to SFO and CN, which promotes the irradiation of the inner surface of the photocatalyst. As shown in Table S2, the total pore volume, average pore size, and specific surface area of SFO, CN, and 7-SFO/CN are provided. It is evident that 7-SFO/CN possesses a larger specific surface area than SFO and CN, which enhances the rapid transfer of reactants and products during the reaction process [31].

XRD was employed to investigate the crystallographic properties of the synthesized materials. As depicted in Figure 2b, the diffraction peaks of SFO exhibited remarkable consistency with standard M-type SrFe_12_O_19_ (JCPDS No. 331340), particularly through characteristic reflections at 30.2° (110), 32.2° (008), 34.1° (107), 37.1° (114), 40.4° (203), 55.2° (205), and 63.1° (2011), which align with previous reports for hydrothermally synthesized analogs [32]. The CN spectrum revealed two distinct peaks at 12.8° and 27.4°, corresponding to the (100) and (002) crystallographic planes of g-C_3_N_4_, respectively. These peaks are associated with the in-plane structure of triazine rings and the interlayer stacking of conjugated aromatic units [33]. Figure S3 provides an enlarged view of Figure 2b, highlighting the changes in the diffraction patterns. As shown, the diffraction intensities of the (100) and (002) crystal planes of g-C_3_N_4_ decreased progressively with increasing SrFe_12_O_19_ content. Notably, the (100) crystal plane became indistinguishable in 9-SFO/CN (Figure S3a), indicating a reduction in the structural order of the triazine rings and a significant alteration in the internal structure of g-C_3_N_4_. Additionally, the diffraction peak of the (002) crystal plane in the composites shifted to higher angles compared to pure CN (Figure S3b). According to Bragg’s law, this shift suggests a decrease in the interlayer spacing of g-C_3_N_4_ due to the incorporation of SrFe_12_O_19_ particles, indicating a stronger interaction between metal ions and heptazine units [15].

The Raman spectroscopy of SFO, CN, and SrFe_12_O_19_/g-C_3_N_4_ composites are presented in Figure 2c. The SFO spectrum exhibits three distinct peaks at 523.1 cm^−1^, 612.6 cm^−1^, and 681.6 cm^−1^. The peak at 523.1 cm^−1^ corresponds to the E2g mode, indicative of mixed octahedral sites. The peaks at 612.6 cm^−1^ and 681.6 cm^−1^ are primarily attributed to the stretching vibrations of iron atoms in octahedral coordination [34]. The CN spectrum displays three characteristic peaks at 707.3 cm^−1^, 751.1 cm^−1^, and 979.5 cm^−1^. The prominent peak at 707.3 cm^−1^ is associated with the condensation of melem into g-C_3_N_4_. The peak at 751.1 cm^−1^ is attributed to the strong Raman signature of melamine and originates from the plane-twisted vibrations of CNC units linked to the triazine ring. The band at 979.5 cm^−1^ is ascribed to the parallel N-bending modes within the triazine rings [35]. For the SrFe_12_O_19_/g-C_3_N_4_ composites, the incorporation of SrFe_12_O_19_ resulted in a noticeable decrease in the intensity of the Raman peaks associated with g-C_3_N_4_, suggesting a reduced proportion of g-C_3_N_4_ within the composite. Given the relatively low content of SrFe_12_O_19_ in the composite material, its characteristic peaks were not clearly discernible. Additionally, the observed shift in the Raman peaks of the composite indicates a significant interaction between SrFe_12_O_19_ and g-C_3_N_4_. These findings collectively confirm the successful synthesis of the SrFe_12_O_19_/g-C_3_N_4_ composite.

The elemental composition and chemical states of SFO, CN, and 7-SFO/CN were analyzed using XPS. The survey spectrum of 7-SFO/CN (Figure 2d) revealed distinct photoelectron signatures of Fe 2p, O 1s, C 1s, and N 1s, confirming the co-existence of these elements. High-resolution analysis of the C 1s spectrum (Figure 2e) resolved two components at 284.80 eV (graphitic carbon, C-C) and 287.95 eV (sp^2^-hybridized C in N=C-N within the triazine rings), respectively [31]. The N 1s spectrum (Figure 2f) was deconvoluted into two peaks at 398.37 eV and 400.53 eV, attributed to N-(C)_3_ (tertiary nitrogen) and C-N=C (sp^2^ hybridized nitrogen), respectively [30]. By integrating the XPS peak areas, the carbon-to-nitrogen (C/N) ratios of CN and 7-SFO/CN were calculated to be 50.5% and 52.3% (Table S3), respectively, indicating the possible presence of nitrogen defects in 7-SFO/CN. These nitrogen defects induce shallow trap states that can capture e^−^ and suppress the deep trapping and direct recombination of photogenerated charges [36]. Furthermore, the ratio of the two peak areas of the N 1s spectrum in CN was 1:0.26, whereas in 7-SFO/CN, this ratio increased to 1:0.3, indicating the formation of nitrogen defects due to the partial absence of N-(C)_3_ groups. The Sr 3d spectrum of SFO (Figure 2g) exhibited photoelectron peaks at 133.15 eV and 134.86 eV, corresponding to the Sr 3d_5/2_ and Sr 3d_3/2_ transitions, respectively. The Sr 3d_5/2_ transition arises from the chemical bond between strontium and oxygen (Sr-O), while the Sr 3d_3/2_ transition indicates the presence of strontium ions (Sr^2+^) [27]. The peaks at 710.29 eV and 723.51 eV were associated with the 2p_3/2_ and 2p_1/2_ of the Fe^2+^, respectively. The peaks at 712.31 eV and 725.07 eV were assigned to the 2p_3/2_ and 2p_1/2_ of the Fe^3+^, respectively (Figure 2h) [37]. Two distinct peaks at 529.71 eV and 532.05 eV in the O 1s spectrum correspond to lattice oxygen in SrFe_12_O_19_ and surface-adsorbed H_2_O, respectively (Figure 2i) [31]. Additionally, almost all peaks in the 7-SFO/CN spectrum exhibited shifts compared to those of SFO and CN, which can be attributed to the heterojunction interaction between SrFe_12_O_19_ and g-C_3_N_4_. This interaction promotes the separation and migration of photogenerated charge carriers, thereby enhancing photocatalytic performance [31,38].

### 3.2. OTC Removal Performance

#### 3.2.1. Comparative Analysis of Oxidation Systems

**Figure 3:**
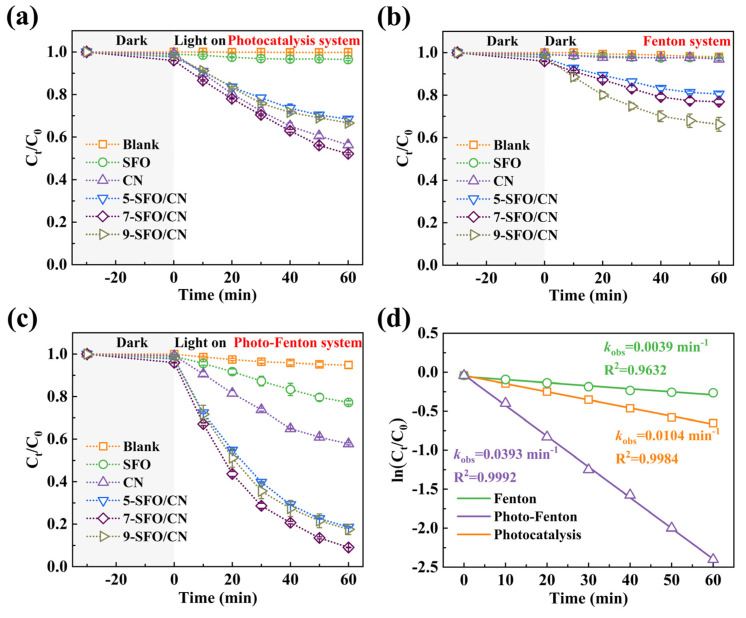
The degradation efficiency of OTC in the (**a**) photocatalysis system, (**b**) Fenton system, and (**c**) photo-Fenton system. (**d**) The rate constants (*k*_obs_) of 7-SFO/CN in different systems. Experimental parameters: [OTC] = 10 mg/L, [Catalyst] = 0.25 g/L, [H_2_O_2_] = 0.1 mL.

The catalytic performance was systematically evaluated through OTC degradation under three operational conditions. Under visible light irradiation alone, OTC did not undergo degradation, whereas SFO exhibited limited activity (3.6% removal in 60 min). CN achieved 43.7% degradation through conventional photocatalysis. The composite series demonstrated composition-dependent performance: 31.6% (5-SFO/CN), 47.9% (7-SFO/CN), and 33.4% (9-SFO/CN) (Figure 3a). The content of SrFe_12_O_19_ emerged as a pivotal factor influencing the degradation performance of the composites. Insufficient SrFe_12_O_19_ content leads to a lack of active sites, while an excessive amount of SrFe_12_O_19_ tends to aggregate, thereby covering some active sites [31]. Notably, the optimal composite, 7-SFO/CN, exhibited a slightly higher degradation rate compared to CN alone, which can be attributed to the formation of a type-I heterojunction between SrFe_12_O_19_ and g-C_3_N_4_. Despite SrFe_12_O_19_ accepting e^−^ and h^+^ transferred from g-C_3_N_4_, its valence and conduction band positions are lower than those of g-C_3_N_4_, resulting in diminished redox capability for SrFe_12_O_19_ relative to g-C_3_N_4_. Figure 3b illustrates the degradation performance of the prepared materials for OTC in the Fenton system (without visible light irradiation). Interestingly, SFO and CN failed to activate H_2_O_2_ for OTC degradation. In contrast, 5-SFO/CN, 7-SFO/CN, and 9-SFO/CN achieved OTC removal efficiencies of 19.5%, 23.1%, and 33.7%, respectively. Although these composites outperformed SFO and CN in the Fenton system, their overall degradation efficiency remained suboptimal. As shown in Figure 3c, under the photo-Fenton system, the OTC degradation efficiencies followed this hierarchy: SFO (22.8%) < CN (42.2%) < 5-SFO/CN (81.4%) < 9-SFO/CN (82.5%) < 7-SFO/CN (90.9%). The optimized 7-SFO/CN composite achieved 90.9% removal efficiency within 60 min, representing a 116% enhancement compared to CN, thus establishing it as the optimal candidate for mechanistic studies. Kinetic analysis in Figure 3d revealed distinct rate constants (*k*_obs_) for 7-SFO/CN across systems: 0.0104 min^−1^ (photocatalysis), 0.0039 min^−1^ (Fenton), and 0.0393 min^−1^ (photo-Fenton), corresponding to pollutant half-lives (t_1/2_) of 66.6 min, 177.7 min, and 17.6 min, respectively. These results clearly indicate that the photo-Fenton system with 7-SFO/CN achieves the highest efficiency in OTC removal. Furthermore, a comparative analysis of OTC removal rates using various catalysts reported in the literature over the past three years (Figure S4) shows that this study achieved the highest OTC removal rate, thereby confirming the superior catalytic performance of 7-SFO/CN [14,39,40,41].

#### 3.2.2. Catalyst Concentration and H_2_O_2_ Dosage

**Figure 4:**
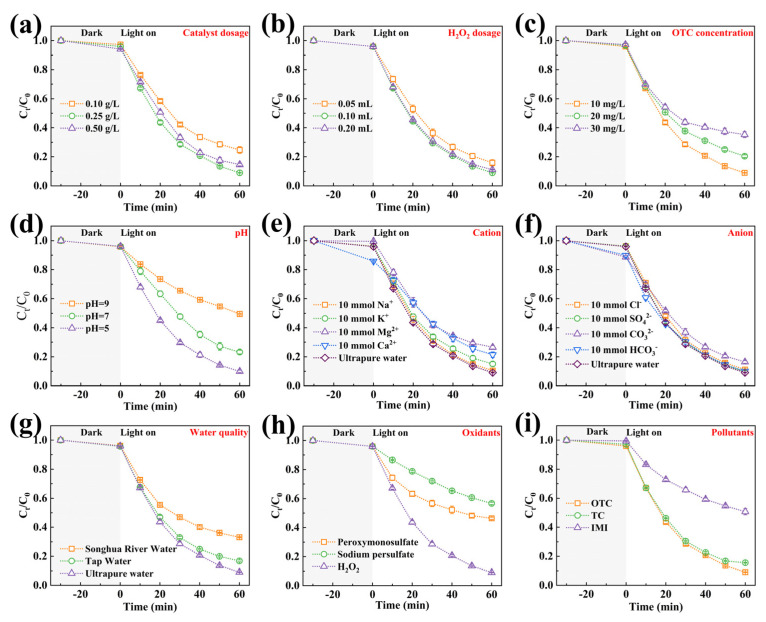
The OTC degradation efficiency of 7-SFO/CN under different conditions: (**a**) catalyst dosage, (**b**) H_2_O_2_ dosage, (**c**) OTC concentration, (**d**) pH value, (**e**) cation, (**f**) anion, (**g**) water quality, and (**h**) oxidants. (**i**) the degradation rate in photo-Fenton system with 7-SFO/CN for different pollutants. Experimental parameters: [pollutant concentration] = 10 mg/L, [Catalyst] = 0.25 g/L, [H_2_O_2_] = 0.1 mL.

The catalyst concentration played a significant role in influencing OTC degradation efficiency (Figure 4a). Specifically, increasing the 7-SFO/CN concentration from 0.1 g/L to 0.25 g/L enhanced OTC removal from 75.3% to 90.9% within 60 min. This enhancement can be attributed to the increased number of reactive sites provided by the higher catalyst dosage, which accelerates both H_2_O_2_ consumption and OTC degradation [30]. However, an excessively high catalyst concentration (0.5 g/L) led to a reduction in efficiency to 85.4%, primarily due to catalyst aggregation and light shielding effects [26]. Optimization of the H_2_O_2_ dosage revealed critical threshold effects (Figure 4b). The degradation rate of OTC increased from 84.1% to 90.9% as the H_2_O_2_ dosage was raised from 0.05 mL to 0.1 mL. Conversely, an excessive H_2_O_2_ dose (0.2 mL) induced free radical scavenging, resulting in a 2.2% decrease in degradation efficiency (Equations (1) and (2)) [40]. These findings underscore the importance of optimizing the stoichiometric ratio of oxidant to catalyst in advanced oxidation processes.

H_2_O_2_ + ·OH → HO_2_· + H_2_O (1)

H_2_O· + ·OH → H_2_O + O_2_ (2)

#### 3.2.3. OTC Concentration and pH Value

Figure 4c illustrates the impact of OTC concentration on the photo-Fenton degradation efficiency of 7-SFO/CN. As the OTC concentration increased from 10 mg·L^−1^ to 20 mg·L^−1^, the degradation efficiency decreased from 90.9% to 79.6%. Further increasing the OTC concentration to 30 mg·L^−1^ resulted in a degradation efficiency of only 64.6%. This reduction in efficiency is likely attributed to the saturation of active sites on the catalyst surface, which occurs due to the excessive presence of OTC and its degradation products in high-concentration solutions [25]. The solution pH significantly modulates OTC degradation efficiency through two primary mechanisms: catalyst surface charge regulation and the thermodynamics of oxidative species generation. The pH value of antibiotic-containing water exhibits considerable variability, which adversely impacts the degradation efficiency of TCs [42]. As illustrated in Figure 4d, acidic conditions (pH = 5) achieved superior OTC removal (90.1%) compared to neutral (76.7%) and alkaline (50.5%) environments. This phenomenon can be attributed to the higher generation of ·OH in acidic environments. In contrast, under neutral or alkaline conditions, H_2_O_2_ decomposition predominantly yields H_2_O and O_2_ [25]. At the same time, we employed the pH drift technique to determine the surface charge characteristics and the point of zero charge (pH pzc) of 7-SFO/CN. As depicted in Figure S5, the pH pzc of 7-SFO/CN was determined to be 7.92. This indicates that the surface of 7-SFO/CN carries a positive charge at pH < 7.92 and transitions to a negative charge at pH > 7.92. OTC is a zwitterionic compound with three dissociation constants (pKa = 3.27, 7.32, 9.11), and its speciation is significantly influenced by the solution pH [11]. Specifically, under acidic, neutral, and alkaline conditions, OTC predominantly exists as positively charged (OTC^+^), neutrally charged molecular species (OTC^0^), and negatively charged (OTC^−^) forms, respectively. In alkaline environments, the electrostatic repulsion between the negatively charged surface of 7-SFO/CN and OTC^−^ further reduces the degradation efficiency [43].

#### 3.2.4. Ions

The ionic composition of aqueous systems significantly influences the modulation of OTC degradation processes. As depicted in Figure 4e, a systematic evaluation of four prevalent cations in aquatic environments highlighted their differential effects on catalytic efficiency. Notably, Na^+^ demonstrated negligible interference with degradation efficiency. Among cations, a distinct inhibitory hierarchy was observed: Mg^2+^ > Ca^2+^ > K^+^. This phenomenon can be attributed to the aggregation of catalyst particles induced by high concentrations of cations, as well as competitive adsorption between divalent cations and Fe^2+^ at the active sites, both of which contribute to reduced catalytic activity [44]. Figure 4f further elucidates the role of common anions (Cl^−^, SO_4_^2−^, HCO_3_^−^, and CO_3_^2−^) in OTC degradation. While Cl^−^, SO_4_^2−^, and HCO_3_^−^ demonstrated negligible effects on degradation efficiency, elevated CO_3_^2−^ concentrations significantly reduced the degradation rate from 90.9% to 83.6%. This suppression mechanism can be attributed to the alkaline microenvironment induced by CO_3_^2−^, which hinders the decomposition of H_2_O_2_ into ·OH radicals [44]. Additionally, CO_3_^2−^ reacts with ·OH to form less reactive ·CO_3_^−^ radicals (Equation (3)) [45]. Notably, the 7-SFO/CN photo-Fenton system successfully degraded over 70% of OTC in water containing high concentrations of coexisting ions, thereby demonstrating robust resistance to ion interference.

CO_3_^2−^ + ·OH → ·CO_3_^−^ + OH^−^ (3)

#### 3.2.5. Water Qualities and Light Sources

The catalytic efficacy of 7-SFO/CN for OTC degradation was systematically investigated across three characteristic aquatic matrices: deionized water, tap water, and Songhua River water. As shown in Figure 4g, the composite exhibited an OTC removal efficiency of 83.2% within 60 min in tap water, despite interference from ions such as Mg^2+^ and Ca^2+^. In contrast, the efficiency decreased to 66.8% in riverine matrices, representing a 24.1% reduction compared to ultrapure water. This performance decline is mechanistically attributed to competitive adsorption between OTC molecules and dissolved organic matter (DOM) present in natural water systems for catalytic active sites [46]. Further insights into the photocatalytic performance were obtained through controlled irradiation experiments (Figure S6). The composite demonstrated superior degradation efficiency under full-spectrum simulated sunlight, achieving an 81.5% removal of OTC within 30 min of reaction time. In contrast, the removal rate decreased to 71.4% under visible light irradiation alone. This enhancement can be attributed to UV-induced photonic activation, as spectral analysis revealed that UV photons generate a higher density of electron–hole pairs than visible light, thereby significantly accelerating redox reactions [47]. Notably, even under conditions of attenuated UV irradiation, the system achieved over 50% OTC degradation, highlighting its exceptional photon utilization capability.

#### 3.2.6. Oxidant Selectivity and Pollutant Degradation Specificity

The oxidant activation capacity of 7-SFO/CN was systematically evaluated by substituting H_2_O_2_ with peroxymonosulfate and sodium persulfate in advanced oxidation processes. As shown in Figure 4h, the composite demonstrated differential activation efficiencies: 53.6% OTC degradation using peroxymonosulfate compared to 43.3% with sodium persulfate, both of which were significantly lower than the 90.9% efficiency achieved through H_2_O_2_ activation. This preference for H_2_O_2_ is further supported by its cost-effectiveness and environmentally friendly decomposition pathway, emphasizing its superiority in photo-Fenton applications [48]. The versatility of the composite for pollutant degradation was further assessed using tetracycline (TC) and imidacloprid (IMI) as model contaminants. The composite exhibited a high TC removal efficiency of 84.4% (Figure 4i), highlighting its efficacy against antibiotic pollutants. Conversely, the degradation efficiency for IMI was only 49.3%, indicating compound-specific challenges potentially arising from the structural resilience of neonicotinoid pesticides [49].

#### 3.2.7. Three-Dimensional Fluorescence Analysis

To further elucidate the degradation process of OTC in the 7-SFO/CN photo-Fenton system, three-dimensional fluorescence analysis technology was used to evaluate the overall changes during OTC degradation. As depicted in Figure S7a, no prominent fluorescence peak was detected in the OTC mother liquor. This can be attributed to the presence of multiple electron-withdrawing groups in OTC, which significantly reduce the fluorescence efficiency of the parent compound. After 5 min of reaction, a fluorescence peak emerged at Ex/Em = 225–275/325–425 nm, corresponding to the fluorescence signal of humic acid-like substances generated by OTC decomposition (Figure S7b). With increasing reaction time, the fluorescence intensity progressively increased, indicating that OTC molecules were continuously decomposed into small organic molecules within the 7-SFO/CN photo-Fenton system (Figure S7c–i) [13]. Additionally, within 60 min, the total organic carbon (TOC) concentration decreased from 4.01 mg·L^−1^ to 3.28 mg·L^−1^, achieving a TOC removal rate of 18.2% in the 7-SFO/CN photo-Fenton system. This suggests that some OTC molecules were mineralized into CO_2_ and H_2_O (Figure S8).

### 3.3. Operational Stability and Magnetic Recoverability

The development of catalysts with sustainable recyclability and maintained catalytic efficacy constitutes a critical prerequisite for the industrial-scale implementation of heterogeneous photo-Fenton systems [48]. Accelerated durability testing revealed only marginal efficiency attenuation, with a 3.1% reduction in OTC degradation efficiency after five successive cycles in the 7-SFO/CN-mediated system (Figure S9). Complementary structural characterization through XRD analysis confirmed the absence of detectable crystalline structure alterations post-cycling (Figure S10), demonstrating exceptional phase stability essential for practical applications. In addition, the concentration of metal ions after the degradation test was quantified by ICP-MS (Table S4). The results show that the metal ion concentration in the 7-SFO/CN solution is significantly lower than that in SFO solution, suggesting that the formation of composite materials effectively suppresses the leaching of metal ions. Meanwhile, the Fe ion concentration in the 7-SFO/CN solution is as low as 0.009 mg·L^−1^, indicating that the circulation of Fe ions plays only a minor role in enhancing catalytic performance.

Unlike nanoscale counterparts, which face inherent recovery limitations and potential ecotoxicological risks [50], the magnetic functionality of 7-SFO/CN addresses these technological challenges. Figure S11 displays the hysteresis loops of CN and 7-SFO/CN. CN shows no magnetic behavior, whereas the incorporation of SrFe_12_O_19_ endows 7-SFO/CN with distinct magnetic properties. As illustrated in Figure S11, CN fails to adhere to the magnet, while 7-SFO/CN is almost completely attached to it. In conclusion, 7-SFO/CN exhibits remarkable stability and magnetic performance, demonstrating significant potential for practical applications.

### 3.4. Photocatalytic Mechanism

#### 3.4.1. Optical Properties and Band Structure

**Figure 5:**
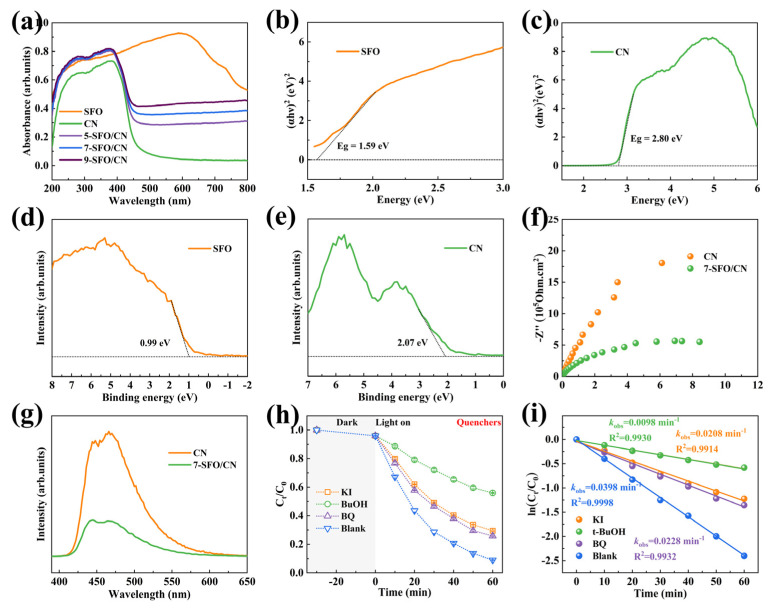
(**a**) UV–vis absorption of as-prepared SFO, CN, and SrFe_12_O_19_/g-C_3_N_4_ composites. Band gap diagram of (**b**) SFO and (**c**) CN. VB_XPS_ of (**d**) SFO and (**e**) CN. (**f**) EIS spectra and (**g**) PL spectra of as-prepared CN and 7-SFO/CN samples. The (**h**) OTC degradation rate and (**i**) rate constants (*k*_obs_) of 7-SFO/CN in the presence of different quenchers.

The optical properties of the photocatalysts were investigated using UV-vis DRS. As illustrated in Figure 5a, the pristine CN exhibits an absorption edge at approximately 500 nm, corresponding to its intrinsic bandgap and indicating a limited response to visible light. In contrast, SFO demonstrates a broad and intense absorption across both the ultraviolet and visible regions, which can be attributed to its unique black surface morphology and narrow bandgap [51]. Notably, the SrFe_12_O_19_/g-C_3_N_4_ composites exhibit progressively enhanced absorption capabilities for both ultraviolet and visible light with increasing SFO content. The bandgap energies of SFO, CN, and SrFe_12_O_19_/g-C_3_N_4_ composites were determined using the Kubelka–Munk equation (Equation (4)):

αhν = A(hν − Eg)^n/2^ (4)

where α, h, ν, A, and Eg represent the absorption coefficient, Planck’s constant, light frequency, proportionality constant, and bandgap energy, respectively. The parameter n denotes the nature of the optical transition, with n = 1 for direct transitions and n = 4 for indirect transitions [10]. The calculated bandgaps for SFO, CN, 5-SFO/CN, 7-SFO/CN, and 9-SFO/CN were determined to be 1.59 eV, 2.80 eV, 2.78 eV, 2.75 eV, and 2.74 eV, respectively (Figure 5b,c and Table S5). Consistent with their optical absorption characteristics, the bandgap of the composite materials decreases with increasing SrFe_12_O_19_ content. To further elucidate the electronic structure, the valence band (VB) positions of SFO and CN were determined using VB-XPS. The VB positions were calculated using the following equation (Equation (5)):

E_VB_ = Φ + VB_XPS_ − 4.44 eV (5)

where E_VB_ represents the valence band position, Φ is the work function of the spectrometer, and VB_XPS_ is the measured binding energy of the valence band maximum [46]. The VB_XPS_ values for SFO and CN were determined to be 0.99 eV and 2.07 eV, respectively (Figure 5d,e). Consequently, the valence band edges were calculated as 0.75 eV for SFO and 1.83 eV for CN. The conduction band positions (E_CB_) were derived using the formula E_CB_ = E_VB_ − Eg, yielding conduction band positions of −0.84 eV for SFO and −0.97 eV for CN. The band structure diagram is shown in Figure S12. These results suggest that the SrFe_12_O_19_/g-C_3_N_4_ heterostructure possesses a well-aligned band structure, facilitating efficient charge separation and transfer under light irradiation.

#### 3.4.2. Enhanced Charge Separation and Transfer

To gain deeper insights into the effects of surface nitrogen defects and heterojunction structures on carrier migration, EIS was employed to investigate the charge transport properties and interfacial resistance of pristine CN and the 7-SFO/CN composite. The smaller arc radius observed in the EIS spectra indicates enhanced efficiency in the separation and migration of photogenerated charges [42]. As shown in Figure 5f, the arc radius of 7-SFO/CN is notably smaller than that of CN, suggesting superior carrier migration efficiency in the composite. To further evaluate the recombination of photogenerated charge carriers, the PL spectra of CN and 7-SFO/CN were recorded under an excitation wavelength of 368 nm. A higher PL intensity corresponds to a faster recombination rate of photogenerated carriers, resulting in reduced photocatalytic activity [10]. As depicted in Figure 5g, the PL peak intensity of 7-SFO/CN is significantly lower than that of CN, confirming that the existence of nitrogen defects and heterojunction structures effectively suppresses the recombination of photogenerated carriers, thereby enhancing the photocatalytic performance.

#### 3.4.3. Free Radical Quenching Experiment

To elucidate the reactive species involved in the photo-Fenton degradation of OTC, radical scavenging experiments were conducted using potassium iodide (KI), tert-butoxide (t-BuOH), and p-benzoquinone (BQ) as scavengers for h^+^, ·OH, and ·O_2_^−^, respectively. As shown in Figure 5h,i, the addition of KI, t-BuOH, and BQ significantly suppressed degradation efficiency of OTC, with the degradation rates decreasing to 70.6%, 44.1%, and 74.2%, respectively. The corresponding *k*_obs_ were calculated as 0.0208 min^−1^, 0.0098 min^−1^, and 0.0228 min^−1^, respectively. These results suggest that ·OH radicals play a predominant role in the degradation process, while h^+^ and ·O_2_^−^ radicals also contribute significantly to photocatalytic activity. To further validate the generation of ROS, EPR spectroscopy was employed. The EPR spectra (Figure S13) clearly demonstrate the presence of ·OH and ·O_2_^−^ radicals in the photo-Fenton system, corroborating the findings from the scavenging experiments. These results collectively highlight the synergistic effects of multiple reactive species in the photo-Fenton degradation of OTC, providing deeper insights into the underlying reaction mechanisms.

**Figure 6:**
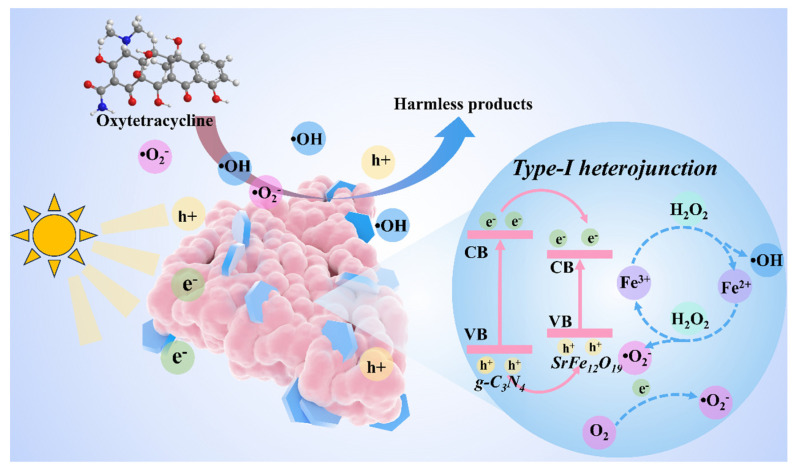
The reaction mechanism of the 7-SFO/CN in photo-Fenton system for degrading OTC.

Based on the radical quenching experiment and the band structure analysis, a detailed photocatalytic reaction mechanism for OTC degradation by the 7-SFO/CN composite is proposed (Figure 6). Under light irradiation, the e^−^ in both SFO and CN are photoexcited from their VB to the conduction bands (CB). Notably, the valence and conduction band positions of SFO are lower than those of CN. According to the second law of thermodynamics and the requirement for Fermi level equilibrium, this energetic alignment drives two simultaneous charge transfer processes: (1) h^+^ migrate from the VB of CN to the VB of SFO, and (2) e^−^ simultaneously transfer from the CB of CN to the corresponding bands of SFO. This charge separation process establishes SFO as the primary reactive site for OTC degradation. The valence band position of SFO (0.75 eV) is insufficient to directly oxidize OH^−^ to generate ·OH, as the thermodynamic requirement for this reaction is 1.99 eV vs. NHE [46]. Therefore, the dominant ·OH generation pathway likely involves the reduction of H_2_O_2_ by e^−^ accumulated on the CB of SFO [52]. Concurrently, the e^−^ in the CB of SFO can reduce dissolved oxygen to produce ·O_2_^−^, as evidenced by the CB position (−0.84 eV) being more negative than the O_2_/·O_2_^−^ redox potential (−0.33 eV vs. NHE) [10]. Furthermore, the h^+^ retained in the VB of SFO exhibits sufficient oxidizing power to directly participate in OTC degradation through hole-mediated oxidation pathways [53]. The proposed reaction mechanism can be summarized as follows:

7-SFO/CN + hv → e^−^ + h^+^ (6)

H_2_O_2_ + e^−^ → ·OH + OH^−^ (7)

O_2_ + e^−^ → ·O_2_^−^ (8)

·OH + ·O_2_^−^+ h^+^ + OTC → degraded products (9)

### 3.5. Degradation Pathways of OTC

**Figure 7:**
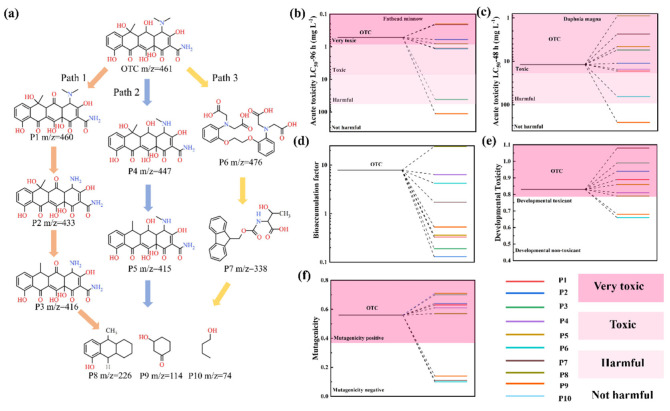
(**a**) Possible degradation pathways of OTC in the photo-Fenton system. (**b**) Fathead minnow LC50 (96 h), (**c**) daphnia magna LC50 (48 h), (**d**) bioaccumulation, (**e**) developmental toxicity, and (**f**) mutagenicity of OTC and its intermediate degradation.

HPLC-MS was employed to identify intermediate products of OTC degradation (Figure S9), revealing three possible degradation pathways (Figure 7a). In Pathway I, direct oxidation by h^+^ and superoxide radicals (·O_2_^−^) initiate sequential transformations: (1) dehydrogenation of OTC (*m*/*z* = 461) forms P1 (*m*/*z* = 460); (2) subsequent demethylation yields P2 (*m*/*z* = 433); and (3) final dehydroxylation produces P3 (*m*/*z* = 416). Pathway II proceeds through N-demethylation of dimethylamine group, generating P4 (*m*/*z* = 447), followed by losing a hydroxyl group at C8 to form P5 (*m*/*z* = 415) [13]. Pathway III involves ·OH radical attacking the aromatic ring of OTC to form an intermediate P6 (*m*/*z* = 476), which is further decomposed into P7 (*m*/*z* = 338) [54]. Ultimately, these intermediates are mineralized into low-molecular-weight compounds, including P8 (*m*/*z* = 226), P9 (*m*/*z* = 114), and P10 (*m*/*z* = 74), through continuous bond scission processes. The toxicity of OTC and its degradation intermediates was evaluated using the Toxicity Estimation Software Tool (T.E.S.T) software (version 5.1.2). As shown in Figure 7b–f, five toxicity indicators were predicted, including Fathead minnow LC50 (96 h), Daphnia magna LC50 (48 h), the bioaccumulation factor, mutagenicity, and developmental toxicity. The results demonstrate significant toxicity reduction across all evaluated indicators, suggesting that the degradation intermediates exhibit lower environmental toxicity compared to the parent OTC compound. Notably, the substantial decrease in bioaccumulation potential underscores the effectiveness of the 7-SFO/CN-based photo-Fenton system in mitigating OTC’s ecological risks.

## 4. Conclusions

In this study, a novel SrFe_12_O_19_/g-C_3_N_4_ composite featuring abundant nitrogen defects was successfully synthesized via a facile one-step co-calcination method, demonstrating exceptional performance for OTC degradation in a photo-Fenton system. Characterization results confirmed that the 7-SFO/CN composite possesses a significantly larger specific surface area compared to both SFO and CN, thereby facilitating the exposure of active sites. Additionally, nitrogen defects at the N-(C)_3_ sites were identified, which effectively inhibited the recombination of photogenerated charge carriers. Mechanistic investigations revealed that the formation of a type-I heterojunction between SFO and CN promotes efficient charge separation, with SFO serving as the primary active site by accepting e^−^ and h^+^ from CN. This charge transfer mechanism significantly enhances the generation of ROS, particularly ·OH radicals, which were identified as the dominant species driving OTC degradation. ·O_2_^−^ and h^+^ also contributed synergistically to the degradation process, as evidenced by radical quenching experiments and EPR analysis. The optimized 7-SFO/CN composite exhibited robust performance under diverse water quality conditions, maintaining high OTC degradation efficiency. Furthermore, the composite demonstrated excellent stability and intrinsic magnetism-based separation capabilities. These attributes, combined with its high efficiency and sustainability, render the 7-SFO/CN-based photo-Fenton system a promising candidate for treating antibiotic-contaminated water. This work not only proposes an environmentally friendly strategy for water remediation but also offers valuable insights into the design of advanced heterojunction photocatalysts for environmental applications.

## Data Availability

Data are contained within the article or Supplementary Material.

## Supplementary Materials

The following supporting information can be downloaded at: https://www.mdpi.com/article/10.3390/nano15110833/s1, Text S1: EIS, HPLC-MS, and EPR characterization details; Text S2: Reaction conditions in degradation experiments; Text S3: Calculation of kinetic parameters for pollutant degradation; Text S4: Three dimensional fluorescence analysis; Table S1: Reagents and materials; Table S2: Total pore volume, average pore size, and specific surface area of SFO, CN, and 7-SFO/CN; Table S3: The C 1s and N 1s peak positions and peak area of CN and 7-SFO/CN; Table S4: The metal ion concentration after the degradation reaction; Table S5: Band gap diagram of SFO, CN, and SrFe_12_O_19_/g-C_3_N_4_ composites; Figure S1: HR-TEM images of 7-SFO/CN; Figure S2: Pore size distribution of (a) SFO, (b) CN, and (c) 7-SFO/CN; Figure S3: Enlarged XRD pattern (a) (10–16°) and (b) (25–30°) of CN and SrFe12O19/g-C3N4 composites; Figure S4: Comparison of the removal rate of OTC by 7-SFO/CN and other catalysts; Figure S5: Zero point charge of 7-SFO/CN; Figure S6: The OTC degradation efficiency of 7-SFO/CN under different light sources; Figure S7: Three-dimensional fluorescence spectra of OTC in photo-Fenton system at different minutes; Figure S8: Total organic carbon (TOC) concentration before and after degradation experiment; Figure S9: Recycle degradation experiment of 7-SFO/CN in photo-Fenton system; Figure S10: XRD patterns of 7-SFO/CN before and after recycle degradation experiment; Figure S11: Hysteresis loops of CN and 7-SFO/CN; Figure S12: Band structure diagrams of SFO and CN; Figure S13: The EPR spectra of 7-SFO/CN for (a) DMPO-·OH and (b) DMPO-·O_2_^−^ in photo-Fenton system; Figure S14: The HPLC-MS spectra of OTC degradation intermediate products.
