# Supplementary material for: Unlocking Synergistic Photo-Fenton Catalysis with Magnetic SrFe12O19/g-C3N4 Heterojunction for Sustainable Oxytetracycline Degradation: Mechanisms and Applications
Source: Nanomaterials (Basel). 2025 May 30;15(11):833. doi: 10.3390/nano15110833 (PMC12157301; doi:10.3390/nano15110833)
Supplement: Supplementary file 1 [file nanomaterials-15-00833-s001.zip › nanomaterials-3606828-supplementary.pdf]

Supplementary Materials

Unlocking Synergistic Photo-Fenton Catalysis with Magnetic $SrFe_{12}O_{19}$/g-C_3N_4 Heterojunction for Sustainable Oxytetracycline Degradation: Mechanisms and Applications

Text Legends

Text S1. EIS, HPLC-MS, and EPR characterization details.

Text S2. Reaction conditions in degradation experiments.

Text S3. Calculation of kinetic parameters for pollutant degradation.

Text S4. Three dimensional fluorescence analysis.

Table Legends

Table S1. Reagents and materials.

Table S2. Total pore volume, average pore size, and specific surface area of SFO, CN, and 7-SFO/CN.

Table S3. The C 1s and N 1s peak positions and peak area of CN and 7-SFO/CN.

Table S4. The metal ion concentration after the degradation reaction.

Table S5. Band gap diagram of SFO, CN, and $SrFe_{12}O_{19}$/g-C_3N_4 composites.

Figure Legends

Figure S1. HR-TEM images of 7-SFO/CN.

Figure S2. Pore size distribution of (a) SFO, (b) CN, and (c) 7-SFO/CN.

Figure S3. Enlarged XRD pattern (a) (10-16°) and (b) (25-30°) of CN and $SrFe_{12}O_{19}$/g-C_3N_4 composites.

Figure S4. Comparison of the removal rate of OTC by 7-SFO/CN and other catalysts.

Figure S5. Zero point charge of 7-SFO/CN.

Figure S6. The OTC degradation efficiency of 7-SFO/CN under different light sources.

Figure S7. Three-dimensional fluorescence spectra of OTC in photo-Fenton system at different minutes.

Figure S8. Total organic carbon (TOC) concentration before and after degradation experiment.

Figure S9. Recycle degradation experiment of 7-SFO/CN in photo-Fenton system.

Figure S10. XRD patterns of 7-SFO/CN before and after recycle degradation experiment.

Figure S11. Hysteresis loops of CN and 7-SFO/CN.

Figure S12. Band structure diagrams of SFO and CN.

Figure S13. The EPR spectra of 7-SFO/CN for (a) DMPO-·OH and (b) DMPO-$\cdot O_2^-$ in photo-Fenton system.

Figure S14. The HPLC-MS spectra of OTC degradation intermediate products.

Text S1. EIS, HPLC-MS, and EPR characterization details.

1. Electrochemical impedance spectra (EIS):

EIS measurements were performed using a Wuhan Coaster CS-350M electrochemical workstation with a three-electrode system. A Pt foil and Ag/AgCl electrode served as the counter and reference electrodes, respectively. The working electrode was prepared by uniformly coating 5 mg of catalyst onto a 1 cm^2 fluorine-doped tin oxide (FTO) glass substrate. The catalyst ink was prepared by dispersing the catalyst in a mixture of 1.8 mL ethanol and 0.2 mL Nafion solution (5 wt%), followed by ultrasonication for 1 h. The electrolyte was 0.2 M Na_2SO_4 aqueous solution, and measurements were conducted at open-circuit potential over a frequency range of 100 Hz to 0.01 Hz with an AC amplitude of 10 mV [1].

2. High-performance liquid chromatography-mass spectrometry (HPLC-MS):

HPLC-MS analysis was performed using a Waters XEVO G2-XS QToF system equipped with a BEH C18 column (50 mm × 2.1 mm, 1.7 μm). The mobile phase consisted of 0.1% (v/v) formic acid in water (A) and acetonitrile (B) at a flow rate of 0.3 mL/min. The gradient elution program was as follows: 0–2 min, 20% B; 2–10 min, 20–80% B; 10–12 min, 80% B; 12–15 min, 80–20% B. The column temperature was maintained at 30°C, and the injection volume was 10 μL. Mass detection was performed in positive electrospray ionization (ESI^+) mode with a capillary voltage of 3.8 kV and a scan range of m/z 50–600.

3. Electronic paramagnetic resonance (EPR):

EPR spectra were recorded on a Bruker A300 spectrometer at room temperature. For

radical trapping, 12.5 mg of catalyst was dispersed in 50 mL of aqueous solution containing 50 mM dimethyl pyridine N-oxide (DMPO) as a spin-trapping agent. After adding 0.1 mL H_2O_2 (30 wt%), the mixture was irradiated under a 300 W xenon lamp ($\lambda \geq 420$ nm) for 5 min. The solution was immediately transferred to a quartz capillary tube for measurement. Spectra were acquired at a microwave frequency of 9.85 GHz, modulation amplitude of 1.0 G, and microwave power of 20 mW.

Text S2. Reaction conditions in degradation experiments.

1. Coexisting ions:

When exploring the effect of coexisting ions in water on the efficiency of 7-SFO/CN photo-Fenton system in degrading OTC, we found through preliminary experiments that the influence of Na^+ and Cl^- on degradation efficiency can be ignored. Therefore, we used OTC solutions containing 10 mmol/L NaCl, KCl, $MgCl_2 \cdot 6H_2O$, and $CaCl_2$ to investigate the effect of cations on degradation efficiency, and OTC solutions containing 10 mmol/L NaCl, Na_2SO_4, Na_2CO_3, and $NaHCO_3$ to investigate the effect of anions on degradation efficiency.

2. Light source:

For the experimental setup, a 300 W xenon lamp without a filter served as the simulated solar light source, while a 5 W ultraviolet lamp provided the ultraviolet light source.

3. Target pollutant:

The ability of the 7-SFO/CN photo-Fenton system to degrade different types of pollutants was investigated using a 10 mg/L TC solution and a 10 mg/L IMI solution. Measure the TC concentration using a UV-vis spectrophotometer at 350 nm. Refer to Dong et al.'s [2] method and use ultra-high performance liquid chromatography to detect IMI concentration. The specific details are as follows: the mobile phase consists of 60% methanol and 40% ultrapure water, with a flow rate of 1.0 $mL \cdot min^{-1}$. The injection volume of IMI solution is 20 μL. The column temperature of the chromatography column is 25°C. The detection wavelength is set to 260 nm.

4. Oxidant:

Add 0.1 ml of peroxymonosulfate (3 mmol·L^{-1}) and sodium persulfate (3 mmol·L^{-1}) instead of H_2O_2 to the reaction system to investigate the ability of photocatalysts to activate different oxidants for the degradation of OTC.

5. Free radical quenching experiment:

To determine the active species in the 7-SFO/CN photo-Fenton system, following the method of SUN et al., t-BuOH (100 mmol/L), BQ (2 mmol/L), and KI (10 mmol/L) were used to capture ·OH, $·O_2^-$, and h^+, respectively [3].

Text S3. Calculation of kinetic parameters for pollutant degradation.

$$\text{degradation rate}=(C_0 - C_t)/C_0 \times 100\% \qquad \text{(S1)}$$

$$\ln(C_t/C_0) = -k_{obs}t \qquad \text{(S2)}$$

$$t_{1/2}=\ln 2/k_{obs} \qquad \text{(S3)}$$

Text S4. Three dimensional fluorescence analysis.

Three dimensional fluorescence testing was used to qualitatively or quantitatively describe the physicochemical properties of organic compounds. In order to gain a deeper understanding of the process of OTC degradation by the 7-SFO/CN photo-Fenton system, three-dimensional fluorescence testing was conducted on OTC degradation solutions with different reaction times. The testing equipment model is Aqualog Horiba from Japan. Specific testing details: Use a 1 cm quartz colorimetric dish to collect excitation wavelength within the range of 200-600 nm. Set integration time to 0.2 seconds, increment to 5 nm, excitation light source to 150 W argon arc lamp.

Table S1. Reagents and materials.

Chemicals	CAS number	molecular formula	Manufacturer
strontium ferrite	12023-91-5	$SrFe_{12}O_{19}$	Xingkaiyue Biotechnology Co., Ltd. Shenzhen, China
melamine	108-78-1	$C_3H_6N_6$	Aladdin Reagent Co. Ltd. Shanghai, China
oxytetracycline	79-57-2	$C_{22}H_{24}N_2O_9$	Aladdin Reagent Co. Ltd. Shanghai, China
hydrogen peroxide	7722-84-1	H_2O_2	Sinopharm Chemical Reagent Co., Ltd. Shanghai, China
sodium hydroxide	1310-73-2	NaOH	Sinopharm Chemical Reagent Co., Ltd. Shanghai, China
hydrochloric acid	7647-01-0	HCl	Aladdin Reagent Co. Ltd. Shanghai, China
sodium chloride	7647-14-5	NaCl	Sinopharm Chemical Reagent Co., Ltd. Shanghai, China
potassium chloride	7447-40-7	KCl	Sinopharm Chemical Reagent Co., Ltd. Shanghai, China
magnesium chloride hexahydrate	7791-18-6	$MgCl_2 \cdot 6H_2O$	Sinopharm Chemical Reagent Co., Ltd. Shanghai, China
calcium chloride	10043-52-4	$CaCl_2$	Sinopharm Chemical Reagent Co., Ltd. Shanghai, China
sodium sulfate	7757-82-6	Na_2SO_4	Sinopharm Chemical Reagent Co., Ltd. Shanghai, China
sodium carbonate	497-19-8	Na_2CO_3	Sinopharm Chemical Reagent Co., Ltd. Shanghai, China
sodium bicarbonate	144-55-8	$NaHCO_3$	Sinopharm Chemical Reagent Co., Ltd. Shanghai, China
Songhua River water	The water sample was collected from the Songhua River section in Harbin.		
tap water	Taken from the laboratory		
ultrapure water	The water sample was prepared using our laboratory's ultra-pure water system.		

Peroxymonosulfate purity≥42%	70693-62-8	$KHSO_5$	Shanghai McLean Biochemical Technology Co., Ltd. Shanghai China
sodium persulfate	7775-27-1	$Na_2S_2O_8$	Sinopharm Chemical Reagent Co., Ltd. Shanghai, China
Tetracycline hydrochloride (TC)	64-75-5	$C_{22}H_{24}N_2O_8 \cdot HCl$	Aladdin Reagent Co. Ltd. Shanghai, China
Imidacloprid (IMI)	105827-78-9	$C_9H_{10}ClN_5O_2$	Altat Technology Co., Ltd. Tianjin, China
potassium iodide (KI)	7681-11-0	KI	Sinopharm Chemical Reagent Co., Ltd. Shanghai, China
tert-butoxide (t-BuOH)	75-65-0	$C_4H_{10}O$	Sinopharm Chemical Reagent Co., Ltd. Shanghai, China
p-benzoquinone (BQ)	106-51-4	$C_6H_4O_2$	Sinopharm Chemical Reagent Co., Ltd. Shanghai, China

Table S2. Total pore volume, average pore size, and specific surface area of SFO, CN, and 7-SFO/CN.

Photocatalyst	Total pore volume	Average pore size	specific surface area
SFO	0.02 cm^3/g	11.14 nm	5.65 m^2/g
CN	0.10 cm^3/g	31.88 nm	12.10 m^2/g
7-SFO/CN	0.12 cm^3/g	26.41 nm	17.96 m^2/g

Table S3. The C 1s and N 1s peak positions and peak area of CN and 7-SFO/CN.

Photocatalyst	CN		7-SFO/CN	
	peak position	peak area	peak position	peak area
sp^2-hybridized C in N=C–N	287.95	254076.76	287.93	221526.12
graphitic carbon, C-C	284.80	72654.77	284.80	67324.32
N-$(C)_3$ of tertiary nitrogen	398.37	515231.24	398.48	425847.82
C-N=C of sp^2 hybridized nitrogen	400.53	132218.10	400.27	126006.14

Table S4. The metal ion concentration after the degradation reaction.

photocatalyst	Fe (mg/L)	Sr (mg/L)
7-SFO/CN	0.009	0.602
SFO	0.028	3.293

Table S5. Band gap diagram of SFO, CN, and $SrFe_{12}O_{19}$/g-C_3N_4 composites.

photocatalyst	Band gap diagram (eV)
SFO	1.59
CN	2.80
5-SFO/CN	2.78
7-SFO/CN	2.75
9-SFO/CN	2.74

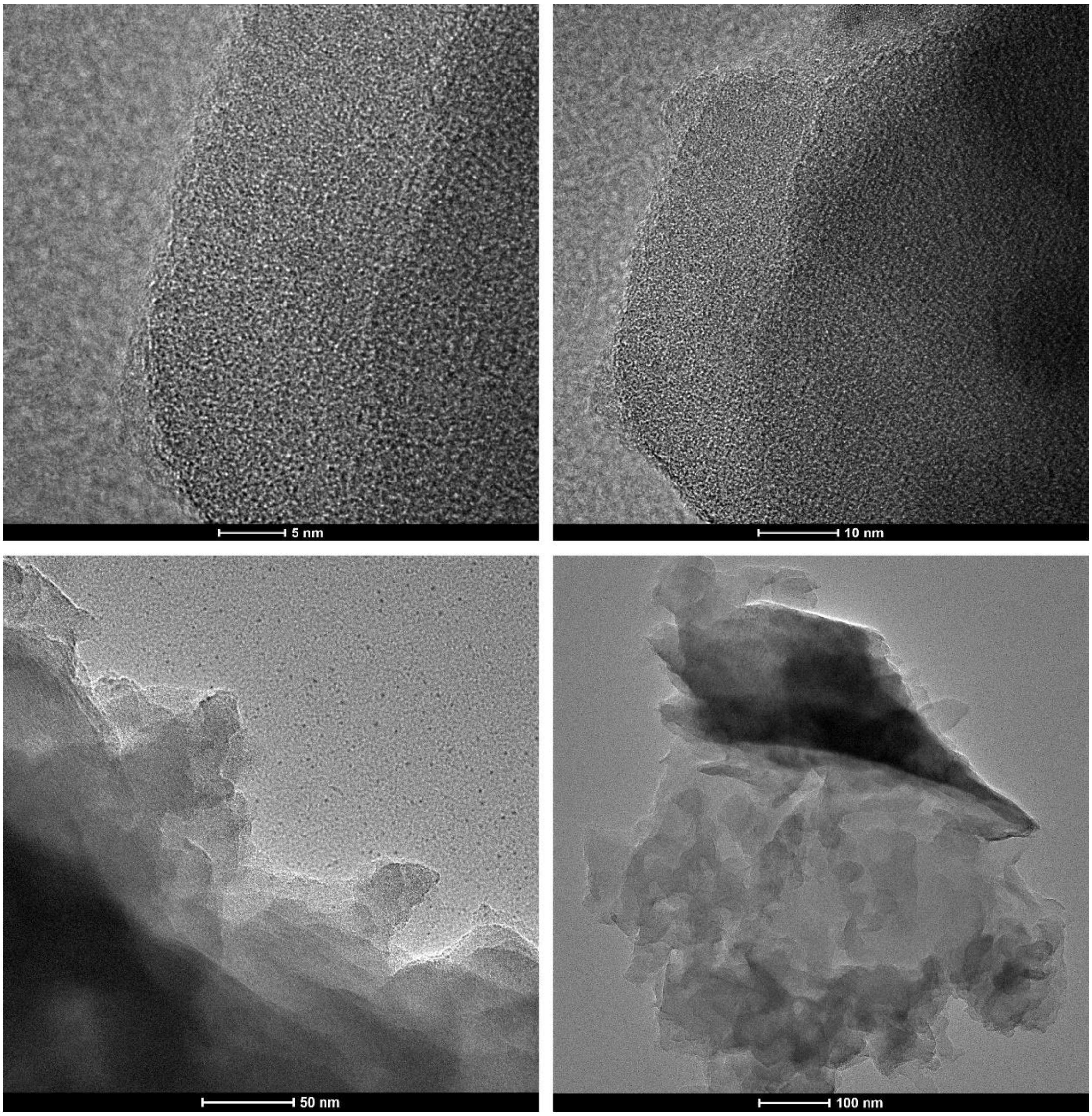

Figure S1. HR-TEM and TEM images of 7-SFO/CN.

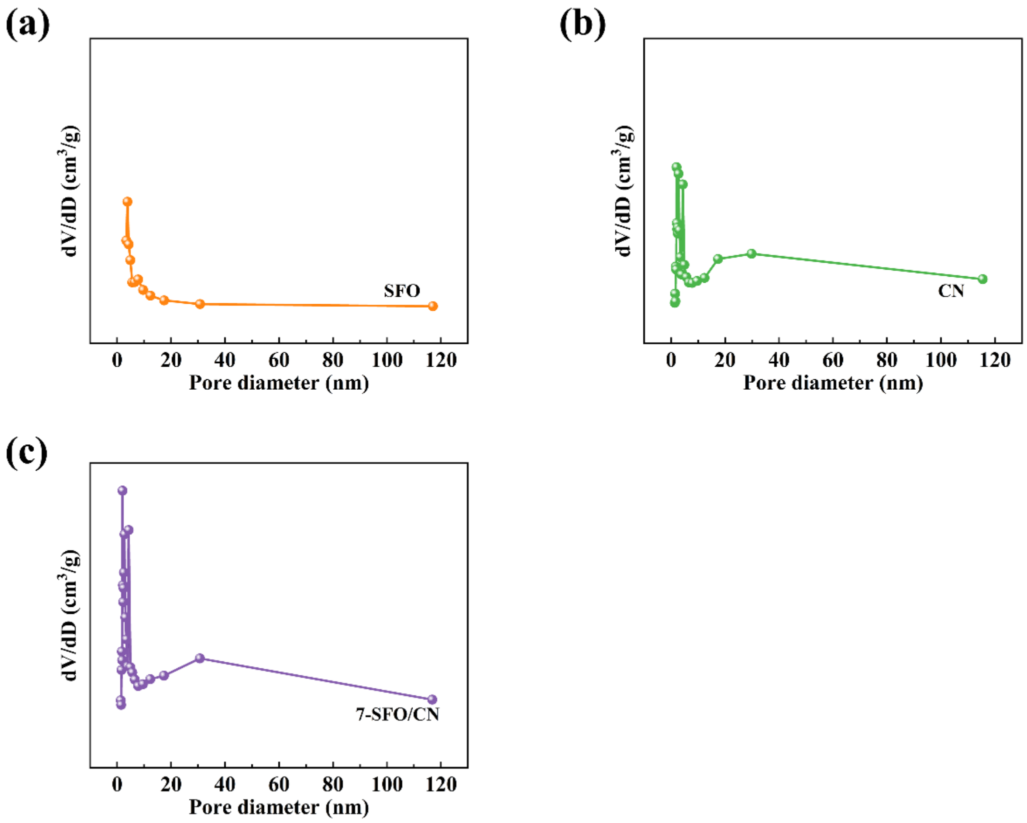

Figure S2. Pore size distribution of (a) SFO, (b) CN, and (c) 7-SFO/CN.

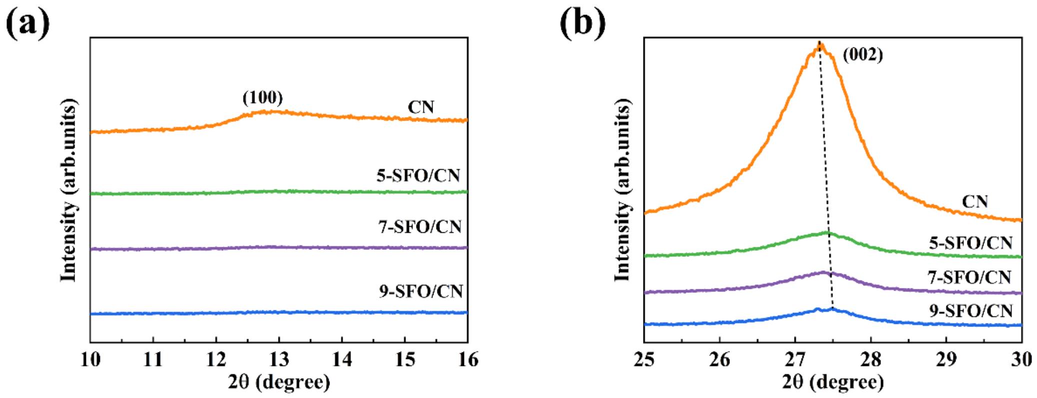

Figure S3. Enlarged XRD pattern (a) (10-16°) and (b) (25-30°) of CN and $SrFe_{12}O_{19}$/g-C_3N_4 composites.

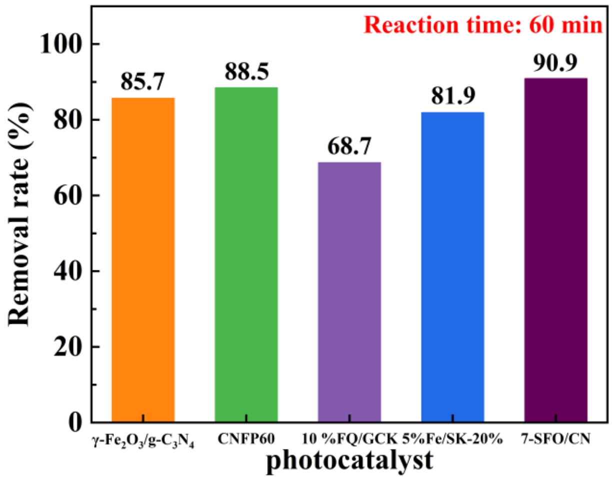

Figure S4. Comparison of the removal rate of OTC by 7-SFO/CN and other catalysts

Comparative literature [4-7]

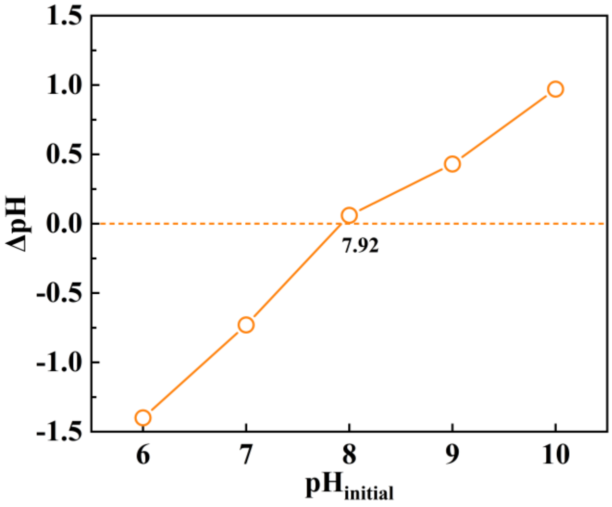

Figure S5. Zero point charge of 7-SFO/CN.

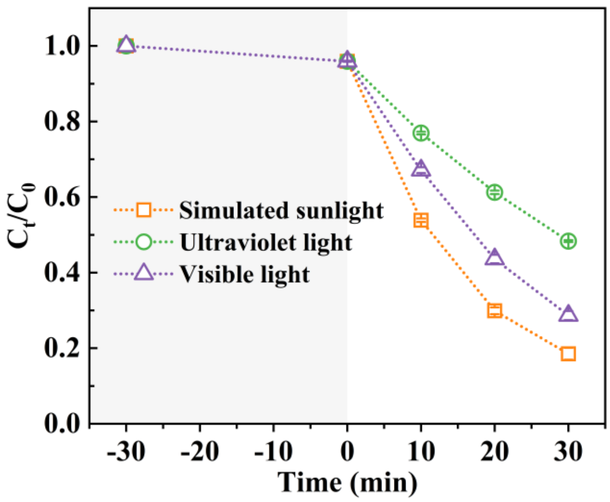

Figure S6. The OTC degradation efficiency of 7-SFO/CN under different light sources.

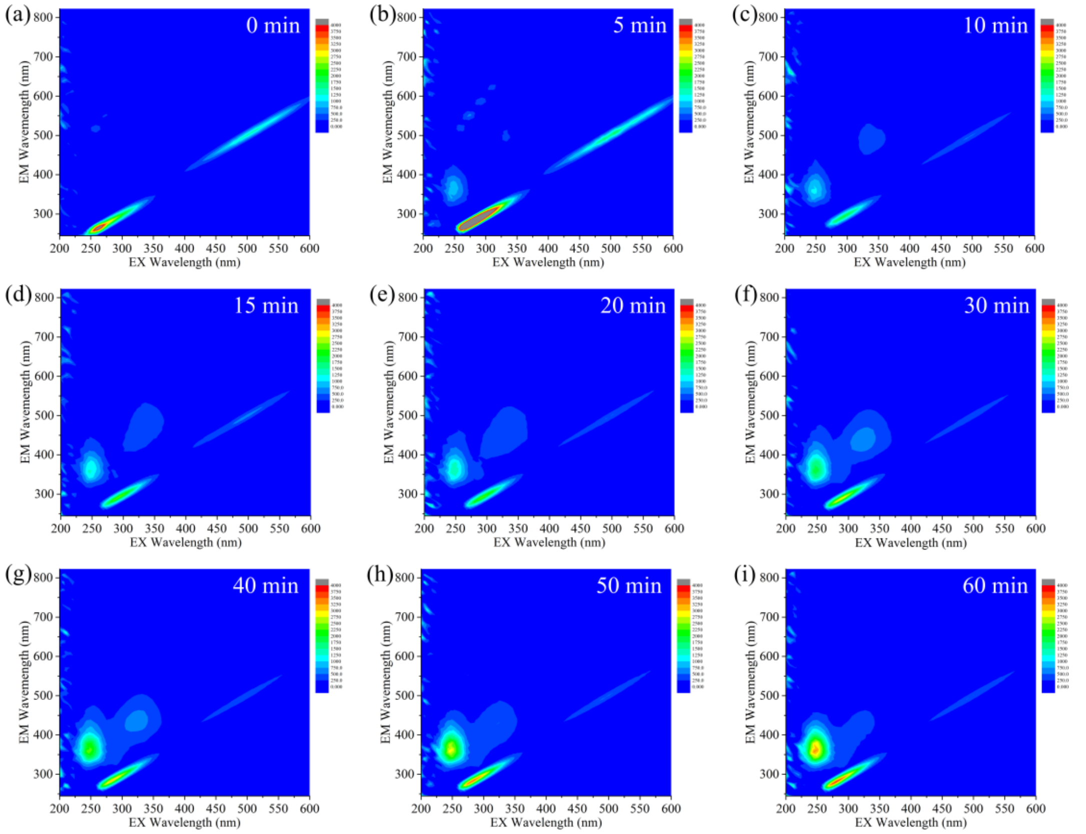

Figure S7. Three-dimensional fluorescence spectra of OTC in photo-Fenton system at different minutes.

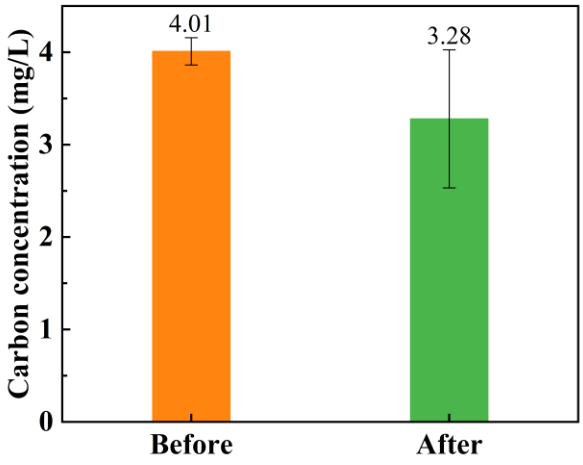

Figure S8. Total organic carbon (TOC) concentration before and after degradation experiment.

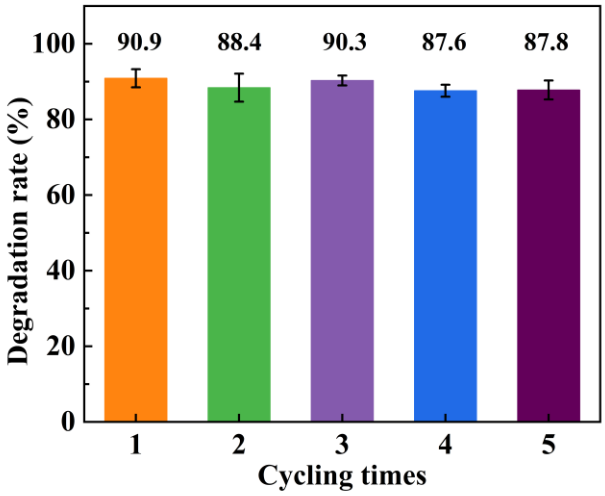

Figure S9. Recycle degradation experiment of 7-SFO/CN in photo-Fenton system.

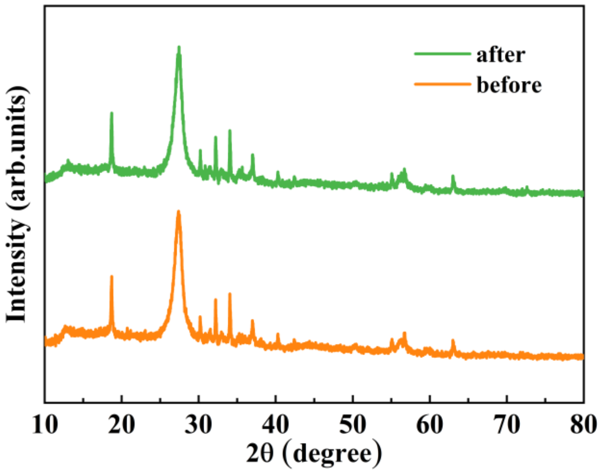

Figure S10. XRD patterns of 7-SFO/CN before and after recycle degradation experiment.

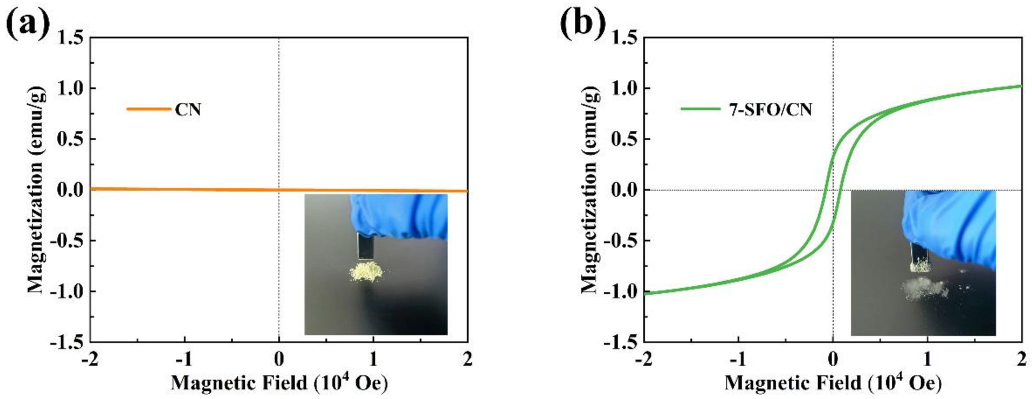

Figure S11. Hysteresis loops of CN and 7-SFO/CN.

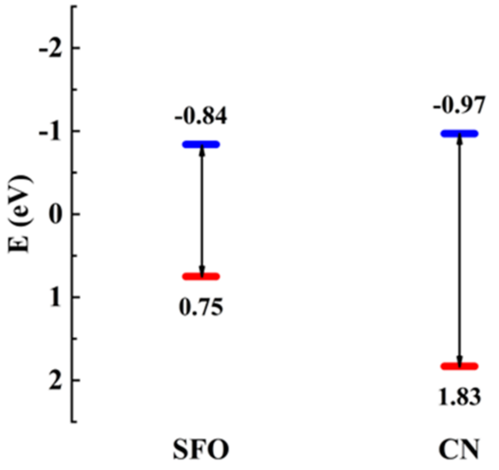

Figure S12. Band structure diagrams of SFO and CN.

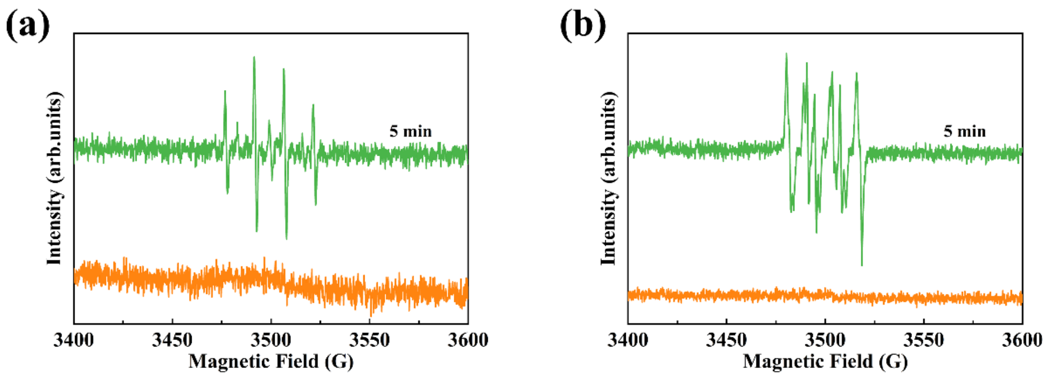

Figure S13. The EPR spectra of 7-SFO/CN for (a) DMPO-·OH and (b) DMPO-·O_2^- in photo-Fenton system.

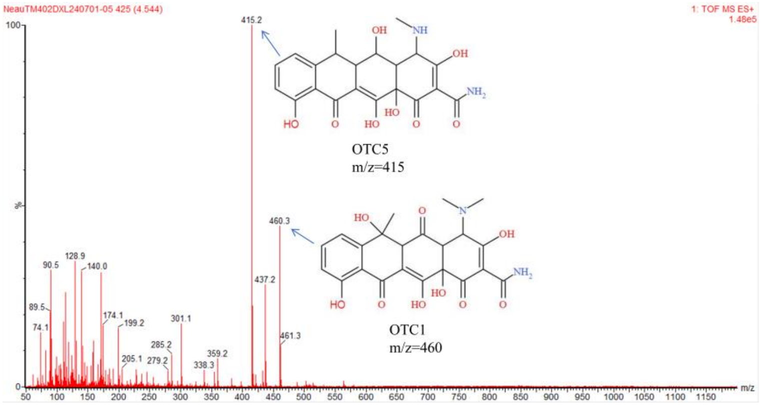
NeauTM402DXL240701-05 425 (4.544)
1: TOF MS ES+
1.48e5
415.2
460.3
437.2
461.3
90.5
128.9
140.0
89.5
74.1
174.1
199.2
301.1
205.1
285.2
279.2
338.3
359.2
OTC5
m/z=415
OTC1
m/z=460
m/z

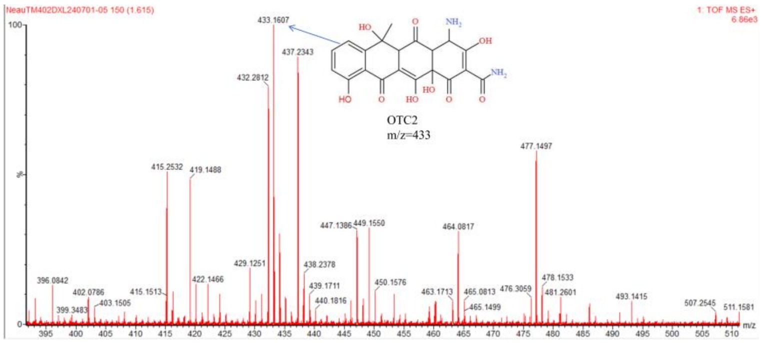
NeauTM402DXL240701-05 150 (1.615)
1: TOF MS ES+
6.86e3
433.1607
437.2343
432.2812
OTC2
m/z=433
477.1497
415.2532
419.1488
447.1386
449.1550
464.0817
429.1251
438.2378
396.0842
402.0786
415.1513
422.1466
439.1711
440.1816
450.1576
463.1713
465.0813
476.3059
478.1533
481.2601
493.1415
507.2545
511.1581
399.3483
403.1505
465.1499
m/z

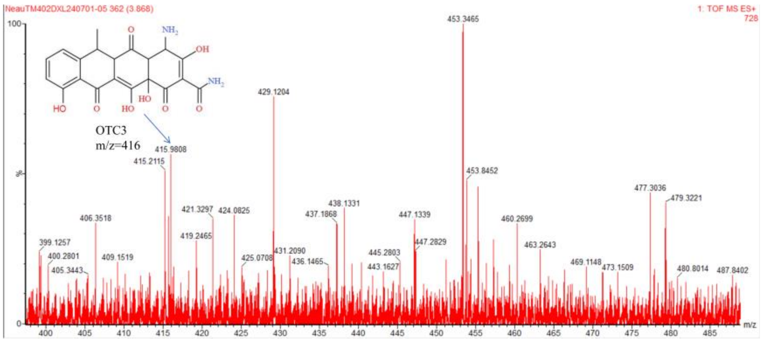
NeauTM402DXL240701-05 362 (3.868)
1: TOF MS ES+
728
453.3465
OTC3
m/z=416
429.1204
415.9808
415.2115
453.8452
477.3036
479.3221
421.3297
424.0825
438.1331
437.1868
447.1339
460.2699
406.3518
419.2465
447.2829
463.2643
399.1257
400.2801
405.3443
409.1519
425.0708
431.2090
436.1465
445.2803
443.1627
469.1148
473.1509
480.8014
487.8402
m/z

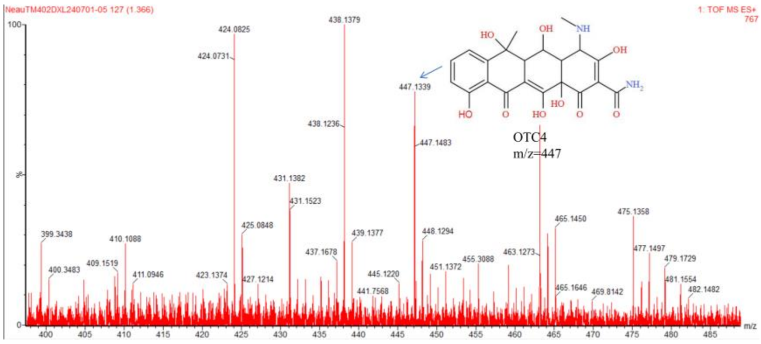
NeauTM402DXL240701-05 127 (1.366)
1: TOF MS ES+
767
OTC4
m/z=447
m/z

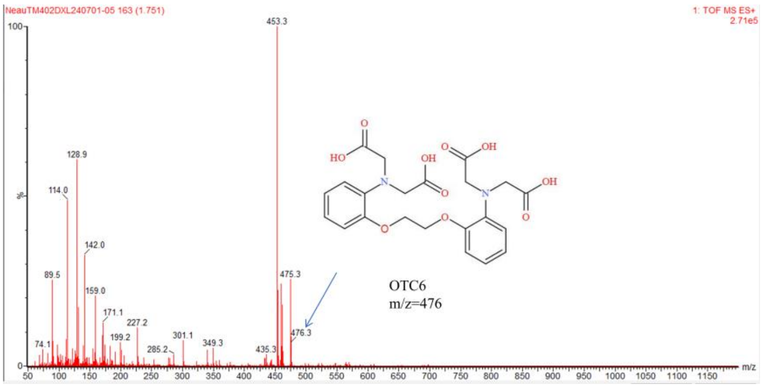
NeauTM402DXL240701-05 163 (1.751)
1: TOF MS ES+
2.71e5
OTC6
m/z=476
m/z

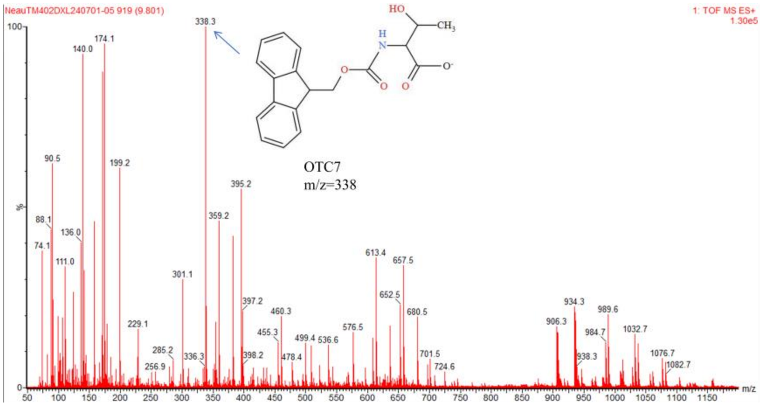
NeauTM402DXL240701-05 919 (9.801)
1: TOF MS ES+
1.30e5
OTC7
m/z=338
m/z

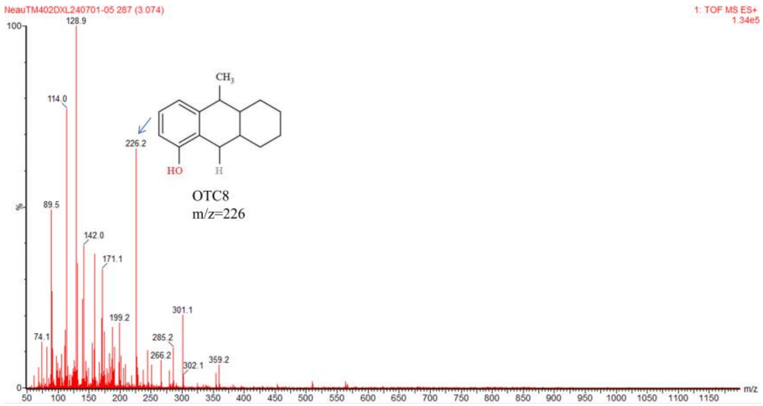

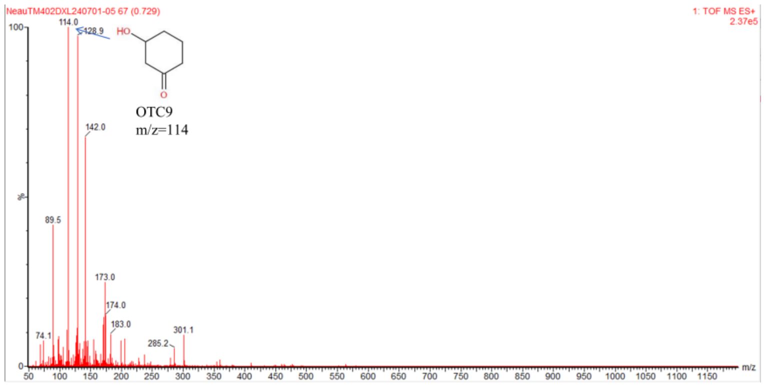

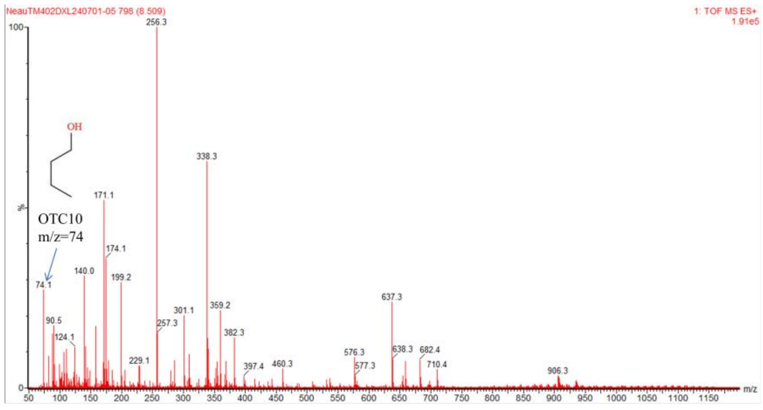

Figure S14. The HPLC-MS spectra of OTC degradation intermediate products.

References

[1] Wang, L.; Ran, X.; Xiao, B.; Lei, L.; Zhu, J.; Xi, X.; Feng, G.; Li, R.; Feng, J. Visible light assisted Fenton degradation of oxytetracycline over perovskite $ErFeO_3$/porous g-C_3N_4 nanosheets p-n heterojunction, *J. Environ. Chem. Eng*. **2022**, *10*, 108330.

[2] Dong, X.; Fu, Q.; Liu, G.; Fan, X.; Zhang, F.; Li, Y.F.; Cui, S. Imidacloprid degradation activated by peroxydisulfate with NiCoAl layered metal oxide catalysts: The unique role of Al, *Sep. Purif. Technol.* **2025**, *356*, 129845.

[3] Sun, H.; Zhou, T.; Kang, J.; Zhao, Y.; Zhang, Y.; Wang, T.; Yin, X. High-efficient degradation of oxytetracycline by visible photo-Fenton process using $MnFe_2O_4$/g-C_3N_4: Performance and mechanisms. *Sep. Purif. Technol*. **2022**, *299*, 121771.

[4] Yang, C., Zhong, H., Deng, J., Li, M., Tang, C., Hu, X., Zhu, M. Z-scheme γ-Fe_2O_3/g-C_3N_4 in Photo-Fenton reaction for oxytetracycline degradation: Mechanism study and DFT calculation. *Sep. Purif. Technol*. **2025**, *354*, 129185.

[5] Xu, F.; Lai, C.; Zhang, M.; Ma, D.; Li, L.; Liu, S.; Zhou, X.; Yan, H.; Wang, N.; Xu, M.; Qin, L.; Yi, H. Graphite carbon nitride coupled with high-dispersed iron (II) phthalocyanine for efficient oxytetracycline degradation under photo-Fenton process: Performance and mechanism. *Sep. Purif. Technol*. **2023**, *308*, 122829.

[6] Qiu, H., Zhu, L., Liu, C., Zhao, M., Li, J., Ding, Z. Fe_3O_4 quantum dots/g-C_3N_4/coal-measure kaolinite composite for efficient photo-Fenton degradation of tetracycline hydrochloride. *Inorg. Chem. Commun.* **2024**, *169*, 113121.

[7] Li, J., Cao, Z., Wang, Q., Cheng, H. Highly dispersed Fe/SnS_2/kaolinite composite for the enhanced photo-Fenton degradation of tetracycline hydrochloride. *J. Alloy.*

Compd. **2024**, *976*, 173061.
